# Coronary microvascular dysfunction: A review of recent progress and clinical implications

**DOI:** 10.3389/fcvm.2023.1111721

**Published:** 2023-01-26

**Authors:** Rajan Rehan, Andy Yong, Martin Ng, James Weaver, Rajesh Puranik

**Affiliations:** ^1^Department of Cardiology, Royal Prince Alfred Hospital, Sydney, NSW, Australia; ^2^Department of Cardiology, Concord Hospital, Sydney, NSW, Australia; ^3^Sydney Medical School, University of Sydney, Darlington, NSW, Australia

**Keywords:** coronary microcirculation, microvascular angina, endothelial dysfunction, inflammation, INOCA

## Abstract

The coronary microcirculation plays a cardinal role in regulating coronary blood flow to meet the changing metabolic demands of the myocardium. Coronary microvascular dysfunction (CMD) refers to structural and functional remodeling of the coronary microcirculation. CMD plays a role in the pathogenesis of obstructive and non-obstructive coronary syndromes as well as myocardial diseases, including heart failure with preserved ejection fraction (HFpEF). Despite recent diagnostic advancements, CMD is often under-appreciated in clinical practice, and may allow for the development of novel therapeutic targets. This review explores the diagnosis and pathogenic role of CMD across a range of cardiovascular diseases, its prognostic significance, and the current therapeutic landscape.

## Introduction

Coronary Microvascular Dysfunction (CMD) is characterized by the remodeling of the coronary microcirculation in response to various pathogenic stimuli. The evolving use of invasive and non-invasive diagnostic modalities has enabled understanding of the role of CMD across a range of cardiovascular conditions, including atherosclerotic epicardial coronary disease, coronary syndromes with no obstructive coronary arteries and primary myocardial pathologies. Furthermore, the presence of CMD is an early marker of cardiovascular disease and is associated with poor clinical outcomes (1). This review aims to describe the mechanistic and prognostic role of CMD across a spectrum of cardiovascular conditions. In addition, we discuss currently available diagnostic modalities to identify CMD, evaluate current treatment strategies and highlight future clinical trials exploring novel therapeutic targets.

## Role of the coronary microcirculation

The coronary arterial bed contains three structurally and functionally distinct compartments (2). The large epicardial coronary arteries (500 μm–5 mm in diameter) function as conduit vessels that offer little resistance to coronary blood flow (CBF), in the absence of obstructive atheroma (3–5). The epicardial pre-arterioles (500–100 μm) account for most of the resistance and respond to flow-related stimuli. The proximal component is more responsive to shifts in flow, whilst the distal is more sensitive to pressure variations (3–5). The intramyocardial arterioles (<100 μm) have the highest resistance and are responsible for the metabolic regulation of CBF in response to myocardial oxygen demand (autoregulation). Lastly, the capillaries (<10 μm) function as exchange vessels, given their large surface area and relatively high permeability (3–5). The coronary microcirculation refers to the epicardial pre-arterioles, intramyocardial arterioles, and capillaries.

Coronary microvascular dysfunction is characterized by structural and functional remodeling of the microcirculation leading to impaired coronary blood flow autoregulation (4). Potential mechanisms include dysfunctional coronary vasodilator capacity and/or enhanced reactivity to microvascular vasoconstriction. The endothelium is fundamental in regulating CBF through the modulation of vasorelaxant substances, including nitric oxide (NO), prostacyclin (PGI2), and endothelium-derived hyperpolarizing factors (EDHF) (6, 7). Dysfunctional endothelium results from pathological vasoconstrictive substances (such as endothelin-1, superoxide, hydrogen peroxide, and thromboxane) overhauling the vascular steady state (8, 9). In addition, microvascular spasm involves the impairment of vasomotion (physiological rhythmical contractions) and is closely linked to the presence of endothelial dysfunction (10, 11). Its complex pathophysiology is characterized by vascular smooth muscle cell (VSMC) dysfunction coupled with a predominance of vasoconstrictive metabolites in the setting of enhanced Rho-kinase activity (12–14). Enhanced Rho-kinase activity results in excessive myosin light chain phosphorylation by inhibiting the myosin-binding submit (MBS), leading to a state of hyper-contractility (15). In addition, chronic low-grade inflammation and overactivity of the autonomic nervous system may also contribute (16, 17).

**FIGURE 1:**
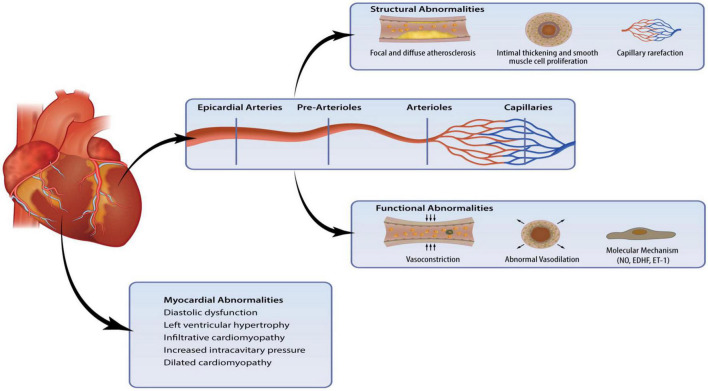
Coronary microvascular dysfunction (CMD) is a result of pathogenic changes in the coronary microcirculation and myocardium. Structural and functional abnormalities lead to endothelial dysfunction and myocardial abnormalities can affect the intramural microvasculature.

The primary structural alternations in CMD include luminal narrowing of the intramural arterioles and capillaries, perivascular fibrosis and capillary rarefaction (4). These pathological changes often occur in the context of left ventricular hypertrophy. In addition, conditions such as hypertrophic cardiomyopathy (HCM) and hypertensive heart disease result in the thickening of medial and intimal vessel walls, leading to impaired CBF (18). Such remodeling of the microcirculation is associated with various comorbidities, including diabetes, hypertension, renal impairment, and diffuse epicardial atherosclerosis (9). Figure 1 summaries the pathogenic abnormalities that characterize CMD.

## Methods of assessing CMD

Assessment of the coronary microcirculation requires functional evaluation of the coronary arteries. Invasive and non-invasive methods can be considered though each has its own advantages and limitations (Tables 1, 2). The optimal method for evaluation depends on patient profile, nature of symptoms (acute vs. chronic), comorbidities, and requirement of serial assessments. Despite growing evidence highlighting the prognostic significance of CMD, European Society of Cardiology (ESC) guidelines currently provide a class IIa recommendation for invasive testing and class IIb recommendation for non-invasive testing (19). In addition, protocols for invasive measurements vary between institutions world-wide, creating difficulties for international comparisons and guidelines.

**TABLE 1:** Non-invasive diagnostic modalities for evaluation of the coronary microcirculation.

Imaging modality	Imaging technique	Substance	Diagnostic threshold	Advantages	Disadvantages
Transthoracic Doppler echocardiography	Pulsed-wave Doppler on the proximal LAD artery	Vasodilators-Adenosine/dipyridamole/ regadenoson	CFRV < 2	– Widely available – Low cost – No radiation exposure.	– Technically difficult hindering reproducibility – Limited to LAD – Obstructive CAD requires prior exclusion
Myocardial contrast echocardiography	Assessment of microbubble velocity and myocardial blood volume	Microbubble contrast agents	MBF < 2	– Widely available – Low cost – No radiation exposure	– Technically difficult hindering reproducibility – Lacking extensive validation CMD – Obstructive CAD requires prior exclusion
Positron emission tomography	Resting and stress perfusion imaging	Radioisotope tracers–^13^Nammonia ^82^Rubidium Vasodilators (as above)	MPR < 2	– Consider reference for non-invasive modalities – All coronary territories evaluated simultaneously	– Expensive – Radiation exposure – Time consuming – Obstructive CAD requires prior exclusion
Cardiac magnetic resonance	Resting and stress perfusion imaging	Gadolinium-based contrast agents Vasodilators (as above)	MPRI < 2	– No radiation – All coronary territories evaluated simultaneously – Excellent spatial resolution	– Expensive – Limited availability – Time consuming – Obstructive CAD requires prior exclusion
Contrast-enhanced cardiac computed tomography	Resting and stress perfusion imaging	Iodine-based contrast agents Vasodilators (as above)	MPR < 2	– Combined assessment of epicardial and microvascular disease – All coronary territories evaluated simultaneously	– Lacking extensive validation for CMD – Radiation exposure – Limited ability for absolute quantification of MBF

CFRV, coronary flow reserve velocity; LAD, left anterior descending; CAD, coronary artery disease; MBF, myocardial blood flow; CMD, coronary microvascular dysfunction; MPR, myocardial perfusion reserve; MPRI, myocardial perfusion reserve index.

**TABLE 2:** Invasive diagnostic modalities for evaluation of the coronary microcirculation.

Imaging modality	Imaging technique	Substance	Diagnostic threshold	Advantages	Disadvantages
Coronary angiography	Serial imaging of coronary arteries whilst passage of angiographic contrast	Iodine-based contrast agents	TIMI–2 TFC > 25frames	– Simple procedure – No additional contrast or radiation use	– Semi-quantitative assessment – Limited sensitivity
Intracoronary thermodilution	Estimation of coronary blood flow using bolus (mean transit time) or continuous thermodilution techniques	Adenosine, Papaverine Saline solutions	CFR_Thermo_ < 2–2.5 IMR > 25	– High success rate of measurements – Validated against clinical outcomes – IMR is specific to microcirculation and not affected by resting hemodynamics	– High intra and inter-observer variability – Varying cut-off values used in clinical studies
Intracoronary Doppler-flow	Direct measure of the coronary peak flow velocity	Adenosine	CFR_Doppler_ < 2.5 HMR > 1.7	– Widely available – Low cost – No radiation exposure.	– Technical complexity HMR cut-off values still debated
Intracoronary vasoreactivity testing	Intracoronary administration of vasoreactive agents	Acetylcholine Ergonovine	ECG Vasocontraction on angiography Symptoms	– Safe and simple method – Does not require additional equipment – Able to assess for epicardial and microvascular spasm	– Additional contrast and radiation – Risk of arrhythmias

TIMI, thrombolysis in myocardial infarction; TFC, TIMI frame count; CFR, coronary flow reserve; IMR, index of microcirculatory resistance; HMR, hyperemic microvascular resistance; ECG, electrocardiography.

### Invasive

Comprehensive functional angiography allows clinicians to investigate the coronary microcirculation and exclude the presence of obstructive CAD (20). Coronary flow reserve (CFR) and measurements of microvascular resistance identify an impaired vasodilatory response (21). In addition, intracoronary provocation testing can identify a microvascular hypercontractive response (22).

Coronary flow reserve is defined as the ratio of maximum hyperemic to basal coronary flow velocity (23). It is a surrogate marker for the vasodilator capacity of the coronary microcirculation and can be assessed *via* an intracoronary Doppler flow wire (CFRDoppl) or temperature sensor-tipped guidewire (CFRthermo) (24). Both techniques have evidence to predict clinical events and can simultaneously measure fractional flow reserve (FFR) to exclude or confirm the presence of hemodynamically significant epicardial stenosis (25, 26). This is crucial as CFR is reduced in patients with epicardial coronary artery disease (CAD) and an impaired hyperemic microvascular dilatory response. CFR is considered normal when values are >2.5 (4, 7). The index of microvascular resistance (IMR) is a marker of minimal achievable resistance of the microcirculation measured using a thermodilution technique (27). It is defined as the product of the distal coronary pressure and mean transit time of a saline bolus during maximal hyperemia. Notably, it is independent of epicardial vascular function, heart rate, blood pressure and ventricular contractility (28). Compared with CFR, IMR provides a more reproducible assessment of the microcirculation, which is independent of hemodynamic perturbations (27). Alternatively, hyperemic microvascular resistance (HMR) using Doppler flow velocity can also be considered.

The diagnosis of coronary spasm (epicardial or microvascular) requires intracoronary provocation testing with acetylcholine (ACh) or ergonovine (ER). Acetylcholine (ACh) is a vasoactive substance that provokes vasospasm *via* cholinergic receptors on vascular smooth muscle cells (29). Healthy endothelium responds to ACh through vasodilatation *via* nitric oxide (NO) release (30). In contrast, dysfunctional endothelium cannot release enough NO to counteract the stimulated muscarinic receptors leading to vasoconstriction (30). In the context of microvascular spasm, the patient will demonstrate ischemic electrocardiographic changes along with reproduction of the typical symptoms (e.g., angina) without angiographic evidence of epicardial vasoconstriction. The safety of intracoronary provocation testing is excellent with similar rates of serious adverse events as routine diagnostic coronary angiography (31, 32). A large retrospective study from Japan of 21,512 patients undergoing pharmacological intracoronary provocation testing demonstrated < 1% serious cardiac complications (33). This was reiterated by Ciliberti et al. who published a systematic review demonstrating a low overall occurrence of major and minor adverse events (0.8 and 4.7%, respectively) (34). Predictors for complications during ACh provocation testing included a history of paroxysmal AF, moderate-to-severe LV diastolic dysfunction and higher QT dispersion at baseline ECG (35). A recent prospective analysis by Montone et al. demonstrated that these complications are not associated with a worse prognosis at a medium-long term follow-up (35). The authors also highlighted the prognostic significance of a positive ACh provocation test, especially in patients with myocardial infarction and no obstructive coronary artery disease (MINOCA) (35).

### Non-invasive

Non-invasive methods to diagnose CMD should only be used after epicardial coronary disease has been excluded. Such techniques can only assess the vasodilator capacity of the coronary microvasculature and provide an estimate of myocardial blood flow. As mentioned above, assessment of endothelial-dependent microvascular dysfunction requires invasive provocation testing.

Transthoracic Doppler echocardiography (TTDE) of the LAD coronary artery is a widely applicable method to assess coronary flow velocity reserve (CFVR) (36). Nevertheless, technical issues, reduced spatial resolution and operator variability limit its use (24). On the other hand, myocardial contrast echocardiography is a promising method that provides a more objective assessment of coronary vasodilator capacity. Its use has also been limited, given the lack of validation in large scale studies (37). Positron emission tomography (PET) and cardiac magnetic resonance imaging (CMR) are two imaging modalities that provide a valid global and regional myocardial blood flow estimate. PET calculates the rate of radioactive tracer uptake into the left ventricular myocardium, allowing for automated quantification of absolute global myocardial blood flow (mL/min/g) at rest and peak hyperemia (38, 39). Alternatively, CMR measures myocardial signal intensity changes between rest and stress images to assess myocardial perfusion reserve (MPR). It can also calculate MPR quantitatively as a ratio of absolute myocardial blood flow (mL/min/g) at peak hyperemia and rest (40). Higher spatial resolution and lack of ionizing radiation make CMR beneficial, though lack of availability and higher costs have limited its use in clinical settings.

## CMD in obstructive and non-obstructive coronary syndromes

### CMD in acute obstructive coronary syndromes

Despite restoring patency of the epicardial coronary vessels in acute coronary syndrome, structural and functional abnormalities of the microvasculature result in suboptimal myocardial reperfusion. This phenomenon, known as coronary microvascular obstruction (CMVO), is an important predictor of left ventricular remodeling, HF, and death in patients with ST-segment elevation acute myocardial infarction (STEMI) (41–43). It’s pathogenesis involves four interacting mechanisms: distal atherothrombotic embolization, ischemia-related injury, reperfusion-related injury, and individual susceptibility (44).

Distal atherothrombotic embolization results in mechanical obstruction and functional impairment *via* biologically active substances promoting a procoagulant state and vasoconstriction (45). Ischemia-related injury is characterized by capillary damage determined by the duration and extent of ischemia (46). Despite restoration of epicardial vessel patency, reperfusion itself can aggravate microvascular injury (MVI) by inducing a vigorous inflammatory response with subsequent leucocyte and platelet plugging, the release of free oxygen radicals and a reduction in nitric oxide bioavailability (47). A subset of patients may develop intramyocardial hemorrhage (IMH), an irreversible pathological consequence of severe MVI (48). Lastly, individual susceptibility to CMVO is characterized by genetic polymorphisms (e.g., 1976T.C mutation of the adenosine 2A receptor gene), diabetes, acute hyperglycemia, hypercholesterolemia, and lack of pre-conditioning (44, 49). Despite its prognostic significance, an optimal treatment strategy for CMVO is still under debate, with numerous RCTs exploring the role of intracoronary thrombolysis illustrating inconsistent results (50–55).

### CMD in chronic obstructive coronary syndrome

The synergistic effect of CMD and obstructive CAD leads to the progression of myocardial ischemia. Epicardial CAD can result in low perfusion pressures distal to the stenosis, which results in abnormal microvascular remodeling and a reduction in coronary vasodilator capacity (56). These pathological changes hinder the development of collateral flow, a critical response to stress-induced myocardial ischemia (56). In addition, the presence of CMD can lead to an underestimation of the degree of epicardial stenosis measured by FFR (57). The prognostic implications of CMD have been highlighted in recent literature (58–60). Van de Hoef et al. demonstrated that an invasively derived CFR < 2.7 in patients with obstructive CAD was associated with increased all-cause mortality (61). Similar findings were derived by Gupta and Cortigiani, who used PET and TTDE, respectively (58, 62). Interestingly, Taqueti et al. confirmed a reduced CFR modified the effect of early revascularization (63). In 329 patients referred for invasive coronary angiography after stress testing, only those with a low CFR appeared to benefit from revascularization, and solely if the revascularization included CABG (63). Hence, CMD in obstructive CAD is not only a prognostic marker but can also guide a successful treatment strategy.

### CMD post-revascularization

Coronary microvascular dysfunction following successful PCI can lead to persistent angina representing a sub-optimal clinical result. The underlying pathophysiology is characterized by distal plaque embolization, neo-atherosclerosis, incomplete revascularization, and vasomotor abnormalities (64, 65). Notably, the pharmacological coating of drug-eluting stents (DES) may enhance coronary vasoconstrictive reactivity and promote neo-atherosclerosis *via* the interaction of the canonical mTOR pathway and FK506-binding protein 12 (66). This concept was emphasized by Ong et al., who illustrated the presence of vasomotor abnormalities in almost half the patients undergoing coronary angiography for recurrent angina after PCI (31). Furthermore, the prognostic significance of CMD post-revascularization was demonstrated by a study of 570 patients that found a correlation between impaired IMR (>25) post-PCI and adverse clinical events in patients with stable CAD (67). This association was independent of the final FFR value suggesting the prognostic significance of CMD despite successful revascularization of epicardial vessels. Likewise, Li et al. and Milo et al. highlighted that a reduction in CFR post-PCI is associated with recurrent angina and abnormal functional stress testing (measured by intracoronary thermodilution and TTDE, respectively) (68, 69). These studies reiterate the clinical significance of residual CMD post-revascularization.

### CMD in ischemia with no obstructive coronary artery disease

Coronary microvascular dysfunction characterizes the mechanistic abnormalities in microvascular angina (MVA), a prevalent endotype in patients with ischemia and no obstructive coronary artery disease (INOCA). CMD is present in almost half of patients with INOCA (70). The abnormal vasomotor function of the coronary microvasculature leads to recurrent angina and a poor quality of life (71). Metabolic risk factors such as, dyslipidemia, obesity and T2DM are highly prevalent in INOCA (72). These comorbidities contribute to endothelial dysfunction by promoting a pro-inflammatory state and increased adrenergic activity.

Coronary microvascular dysfunction is an important prognostic marker for this cohort of patients. In the WISE study, a prospective cohort study of women with symptoms of ischemia undergoing coronary angiography, reduced CFR was a powerful incremental predictor of MACE (73). Likewise, subsequent studies using non-invasive measures of coronary vasodilatory capacity demonstrated similar results (62, 74). Murthy et al. provided the most definitive data highlighting the association between CFR < 2.0 (measured by PET) with a 7.8 and 5.6% annualized rate of MACE among symptomatic men and women with INOCA (compared to 3.3 and 1.7%, respectively, for CFR ≥ 2.0) (75).

Classifying patients into specific INOCA endotypes (microvascular angina, epicardial vasospastic angina, non-cardiac chest pain) can allow clinicians to prescribe targeted pharmacological treatment. For example, the CorMica trial demonstrated that functional angiography for correct classification of INOCA endotypes leads to improved patient outcomes, including a reduction in angina severity and better quality of life (20).

### CMD in myocardial infarction with no obstructive coronary artery disease

Myocardial infarction with non-obstructive coronary artery disease (MINOCA) accounts for approximately 6–14% of patients with acute MI cases and represents a myriad of underlying etiologies (76). These patients have a 1-year mortality and rehospitalization rates comparable to those with MI and obstructive coronary artery disease (MI-CAD) (77, 78). Despite the poor understanding of the underlying pathophysiological mechanism in MINOCA, over 20% of cases are associated with CMD (79). CMD develops due to two separate mechanistic pathways: acute hyper-adrenergic tone with a rapid rise in microvascular resistance leading to cardiomyocyte death or chronic microvascular ischemia coupled with an acute trigger causing myocardial necrosis. The prognostic significance of CMD in patients with MINOCA was demonstrated by Abdu and colleagues’ evaluation of 109 patients (80). Microvascular function was assessed *via* coronary angiography-derived index of microvascular resistance (caIMR), a novel non-invasive method based on quantitative flow ratio (QFR). Over half (50.5%) of the patients were classified into the high caIMR group, and the rate of MACE was significantly higher in this cohort (80). In some cases, CMD may not be the sole cause of MINOCA but simply an “innocent” bystander playing a pathogenic role. For example, using stress CMR, Mauricio et al. demonstrated abnormal stress perfusion in 63% of females with MINOCA, however, this only corresponded with a myocardial scar in 75% of cases (81). These findings suggest that further mechanistic studies are required to elucidate the precise contribution of CMD in MINOCA.

## CMD in myocardial pathologies

### CMD in heart failure with preserved ejection fraction

Heart failure with preserved ejection fraction (HFpEF) refers to a heterogeneous syndrome characterized by diastolic dysfunction secondary to adverse LV remodeling, cardiometabolic dysfunction, and extracellular fibrosis (82–84). In recent times, our understanding of this clinical entity has evolved. We now acknowledge HFpEF is a systemic disease characterized by inflammation and microvascular endothelial dysfunction. It is hypothesized that the interplay between immunoregulatory cytokines and reduced NO results in an inflammatory milieu leading to CMD (85). Comorbidities such as hypertension, type II diabetes, chronic kidney disease, and obesity contribute to and accelerate this process, ultimately leading to adverse clinical outcomes (86).

Over the last decade, research has provided insights to the links between CMD and HFpEF. The PROMIS-HFpEF study demonstrated the presence of CMD (as per Doppler echocardiography) in 75% of HFpEF patients and its association with markers of HF severity (87). In addition, a recent cohort study confirmed similar findings in 106 patients who underwent functional angiography and CMR. Overall, 85% had evidence of CMD, with the majority illustrating endothelial-independent dysfunction (88). These results are consistent with older retrospective studies that suggested this association (87, 89). Taqueti et al. not only confirmed the presence of CMD in HFpEF but highlighted its prognostic significance. CMD was associated with a > 5-fold risk of HFpEF hospitalization, higher rate of MACE and mortality (90). Despite the overwhelming evidence confirming the presence of CMD in HFpEF, the question of cause or effect remains. Does CMD lead to HFpEF, or does myocardial remodeling in HFpEF lead to coronary microvascular ischemia and subsequent dysfunction? Nonetheless, targeting CMD in HFpEF patients might be a promising therapeutic strategy in years to come.

### CMD takotsubo cardiomyopathy

Takotsubo Cardiomyopathy (TTC) is an acute and reversible form of myocardial dysfunction. Despite several etiopathogenetic mechanisms proposed, most believe it is characterized by a catecholaminergic surge following an emotional or psychological stressor leading to coronary microvascular vasoconstriction (91). Numerous diagnostic indices have confirmed the presence of CMD in this syndrome. Non-invasive modalities demonstrated reduced myocardial blood flow and perfusion defects within dysfunctional segments (92–94). In addition, numerous groups confirmed an elevated IMR in such patients, suggesting impaired microcirculatory resistance (95–97). The degree of CMD may have prognostic significance in this cohort. Montone et al. prospectively evaluated 101 patients and demonstrated a correlation between coronary slow flow on angiography and poor long-term clinical outcomes (98). Such measures may allow clinicians to risk stratify patients and consider aggressive medical therapy with close follow-up.

### CMD in miscellaneous cardiomyopathies

Hypertrophic cardiomyopathy (HCM) is a genetic disorder that involves a complex interplay of myocyte disarray, interstitial fibrosis, and coronary microvascular remodeling (84, 99). In the presence of increased oxygen demand, such structural changes predispose the myocardium to ischemia leading to clinical sequelae of angina, syncope, and sudden death (100). Multiple coronary microvascular indices have demonstrated the presence of CMD in HCM. Camici et al. used PET to quantify MBF and demonstrate the presence of CMD in both hypertrophied and non-hypertrophied myocardium (101). Further studies highlighted a reduced CFR in the sub-endocardium secondary to elevated extravascular compressive forces. The presence of CMD in HCM has important prognostic implications. Cecchi et al. found that the severity of CMD (as assessed by PET) was an independent predictor of cardiovascular morbidity and mortality (102). Moreover, they highlighted that severe CMD was even present in many asymptomatic patients, preceding adverse cardiovascular outcomes by several years.

Infiltrative cardiomyopathies represent a group of disorders characterized by changes in the myocardium and coronary microvasculature leading to significant remodeling and mechanical dysfunction. Over the past decade, clinical research has confirmed the presence of CMD in cardiac amyloidosis, Anderson-Fabry disease and sarcoidosis. Contemporary literature employing non-invasive imaging demonstrated a significant reduction in hyperemic blood flow and reserve in this cohort (103–105). Interestingly, the presence of CMD can also be an early marker of disease activity and may denote a favorable window for an aggressive therapy (104, 106). Despite these efforts, there is minimal literature available to explain the prognostic impact of CMD in these patients.

## Treatment and future directions

**FIGURE 2:**
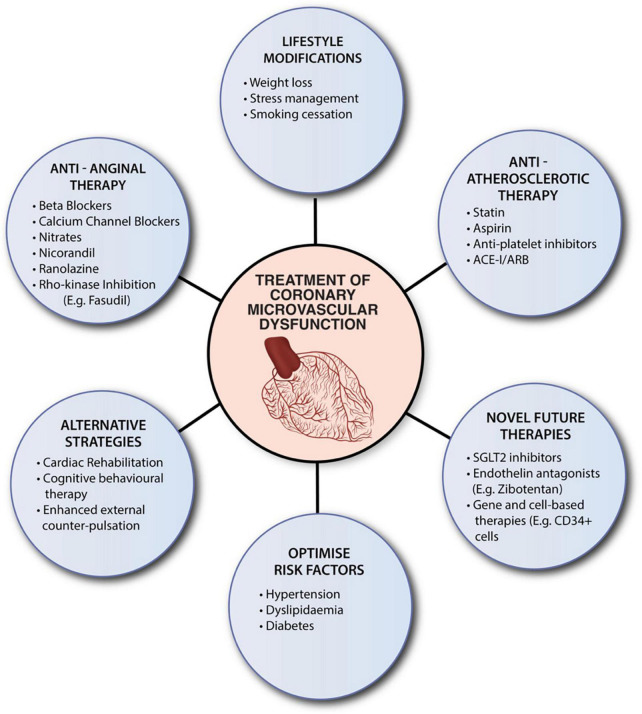
Summaries current therapeutic strategies for CMD. Despite the absence of specific evidenced-based therapies, clinicians should consider optimizing risk factors, encourage lifestyle modifications, and utilize conventional pharmacological therapy. ACE-1, angiotensin-converting enzyme inhibitors (ACE-I); ARB, angiotensin receptor blockers; SGLT2, sodium-glucose cotransporter-2 inhibitor.

At this stage, there is no specific therapy for CMD. Current practice is primarily based on studies with heterogeneous populations and varied diagnostic techniques. Current therapeutic strategies focus on optimizing risk factors, lifestyle modifications, and conventional pharmacological therapy (Figure 2). Risk factors for CMD overlap with traditional cardiac risk factors. Management of diabetes, hypertension and hypercholesterolemia is essential in this cohort. In addition, lifestyle modifications, including physical exercise, smoking cessation and weight loss, contribute to improvements in cardiorespiratory fitness and CVD outcomes (107–110). Recently, Camilli et al. demonstrated an association between ambient air pollution and coronary vasomotor abnormalities (111). In particular, higher levels of airborne particular matter (PM) < 2.5 μm was an independent risk factor for the occurrence of epicardial spasm and MINOCA (111). These findings suggest that reducing air pollution levels may improve ischemic symptoms.

Conventional pharmacotherapy includes anti-atherosclerotic and anti-anginal and drugs. Anti-atherosclerotic therapy consists of angiotensin-converting enzyme inhibitors (ACE-I) or receptor blockers (ARB), statins and thromboxane A2 inhibitors (TXA2). ACE-I elicit vasoprotective effects through inhibition of the renin-angiotensin axis. The TREND data (Trial on Reversing Endothelial Dysfunction) demonstrated the ability of ACE-I, particularly quinapril, to reverse endothelial dysfunction (112). Subsequent studies have emulated similar results for ARB (113). Statins are primarily HMG-CoA reductase inhibitors that reduce plaque, macrophage and foam cell formation. Their off-target anti-inflammatory effects play a prominent role in CMD (114). TXA2 inhibitors, such as aspirin, minimize platelet-rich-microemboli, which confers microvascular protection against oxidative injury. Together, these agents’ anti-inflammatory and antioxidant properties improve myocardial perfusion and coronary endothelial function.

Regarding anti-anginal pharmacotherapy, beta-blockers are first line for patients with CMD. They primarily improve symptoms of effort angina in patients with elevated adrenergic activity. Notably, third-generation beta-blockers (e.g., nebivolol and carvedilol) can reduce myocardial oxygen demand through their adjunctive NO-mediated vasodilatory effect (115). Short-acting nitrates can also be considered in patients with anginal attacks with an abnormal vasodilatory reserve (116). On the other hand, non-dihydropyridine calcium-channel blockers are preferred in patients with vasospasm-mediated CMD (116). Ranolazine and Fasudil are novel agents that may provide concurrent benefits. Ranolazine is a piperazine derivative that selectively inhibits the late sodium current and reduces intracellular calcium in cardiomyocytes, leading to improved ventricular relaxation and facilitating microvascular function (117). Small RCT’s have demonstrated varied efficacy, though patient stratification *via* invasive CFR may identify a beneficial population for this agent (118, 119). Fasudil is a specific Rho-kinase inhibitor highly effective in preventing ACh-induced coronary spasm (120, 121). The Rho-kinase pathway is involved in endothelial dysfunction, vasospasm and inflammatory cell accumulation. Fasudil can target these pathogenic mechanisms leading to a reduction in myocardial ischemia and symptoms of angina (120, 121).

Fortunately, multiple randomized controlled trials are underway to elucidate specific therapies for CMD. For example, the WARRIOR trial (Women’s Ischemia Treatment Reduces Events In Non-Obstructive CAD–NCT03417388) is a prospective, randomized trial evaluating intensive medical therapy (high-intensity statin, ACE-Is or ARBs, and aspirin) vs. usual care in 4,422 symptomatic women with INOCA (122). Furthermore, a randomized controlled trial (RCT) assessing Zibotentan (NCT04097314), a potent and selective antagonist of Endothelin-A, in MVA is near completion (123). As previously mentioned, CMVO is an essential predictor of left ventricular remodeling, HF, and death in patients with STEMI. The RESTORE-MI (Restoring Microcirculatory Perfusion in STEMI) trial is currently recruiting patients with STEMI and objective evidence of microvascular dysfunction (IMR value > 32) following reperfusion. This multi-center RCT assesses the efficacy of intracoronary tenecteplase, a fibrin specific thrombolytic, as a novel therapy for these patients.

Sodium-glucose Cotransport-2 Inhibitors (SGLT2i) is another drug class that may show hope for patients with CMD. Empagliflozin was shown to restore the beneficial effect of cardiac microvascular endothelial cells on cardiomyocyte function and demonstrate an improvement in CFVR in a prediabetic mouse model (124). Furthermore, the positive results of both the EMPEROR-PRESERVED (Empagliflozin Outcome Trial in Patients with Chronic Health Failure with Preserved Ejection Fraction) and DELIVER (Dapagloflozin Evaluation to Improve the LIVEs of Patients with Preserved Ejection Fraction Heart Failure) trials highlight the need for further studies on a homogenous patient population with CMD (125, 126).

Concentrating on MINOCA, current prospective trials focus on secondary preventive treatments and precision therapy. The MINOCA-BAT (Randomized Evaluation of Beta Blocker and ACEI/ARB Treatment in MINOCA Patients) trial aims to determine whether beta-blocker and/or ACE-I/ARBs reduce the composite end-point of all-cause mortality, readmission for MI, ischemic stroke or heart failure (NCTO3686696) (127). Likewise, the StratMEd-MINOCA (Stratified Medicine of Eplerenone in Acute MI/Injury) trial will assess if a stratified medical approach with early risk stratification by CMD (defined as IMR ≥ 25) coupled with mineralocorticoid antagonist therapy will limit myocardial damage (NCT05198791). Lastly, the PROMISE trial (Prognostic Value of Precision Medicine in Patients With MINOCA) will evaluate whether a precision-medicine approach with a specific therapy tailored to the underlying pathogenic mechanism will improve the quality of life in MINOCA patients (128).

## Summary and perspectives

Coronary microvascular dysfunction is prevalent across various cardiac conditions and characterized by structural and functional abnormalities of the coronary microcirculation. Its pathogenic role in myocardial ischemia contributes to clinical manifestations across these disorders and is associated with an adverse prognosis. Notably, the presence of CMD is often detectable prior to clinical symptoms and may support the potential for early risk stratification and treatment. Unfortunately, despite significant advances over the past decade, clinicians do not have a specific agent for treating this condition. Clarifying the mechanistic role of CMD across clinical phenotypes and identification of novel therapeutic targets requires further research. In line with ongoing RCTs, developing personalized treatments across this heterogenous cohort is the next challenge in cardiovascular medicine.

## Author contributions

RR, AY, JW, and RP: conceptualization. RR and RP: methodology. RR: investigation, resources, and writing—original draft preparation. RR, AY, MN, JW, and RP: writing—review and editing. JW and RP: supervision. All authors have read and agreed to the published version of the manuscript and fulfill relevant criteria for authorship.
